# Professional development session for early career scientists at SITC 2012

**DOI:** 10.1186/2051-1426-1-6

**Published:** 2013-06-27

**Authors:** Christian M Capitini, William L Redmond, Kimberly A Shafer-Weaver

**Affiliations:** 1 Department of Pediatrics and UW Carbone Cancer Center, University of Wisconsin School of Medicine and Public Health, Madison, WI 53705, USA; 2 Earle A. Chiles Research Institute, Robert W. Franz Cancer Research Center, Providence Portland Medical Center, Portland, OR 97213, USA; 3 National Institute of Allergy and Infectious Diseases, National Institutes of Health, Bethesda, MD 20892, USA

**Keywords:** Professional development, Academia, Industry and government positions, K awards, Tenure process, Business of research

## Abstract

The Society for Immunotherapy of Cancer (SITC) 2012 Professional Development Session was held as part of the SITC 27th Annual Meeting, Washington, DC, on October 24, 2012. The session was designed as a new opportunity for early career investigators to learn about relevant career development topics in a didactic setting.

## Background

The Society for Immunotherapy of Cancer (SITC) 2012 Professional Development Session was held as part of the SITC 27th Annual Meeting, Washington, DC, on October 24, 2012. Experts from academia, industry and government were available to discuss 3 main topics – obtaining a K award from the National Institutes of Health (NIH), preparing for tenure and the business of research. In addition, the session concluded with a career panel that outlined opportunities in academia, industry and government. With integrated breaks for questions and networking, attendees were also able to closely interact with the invited experts.

SITC is a non-profit group of medical professionals established in 1984 to facilitate the exchange and promotion of scientific information about the promise and breakthroughs of immunotherapy for cancer patients. Society members include a constituency of nearly 600 clinical and basic scientists from around the world working in academia, industry, and governmental regulatory agencies. SITC’s members represent 17 medical specialties and are engaged in research and treatment of most types of cancer. The Society was founded on the belief that new immune-based treatments would continue to complement traditional cancer treatments and move into the mainstream in the fight against cancer. To aid in this effort, SITC provides a venue to facilitate the discussion of current clinical trial results and methodologies, as well as means to collaborate on new initiatives in tumor immunology and cancer immunotherapy with the ultimate goal of improving cancer patient outcomes.

### Getting a K99/R00 award from the NIH

Shari Pilon-Thomas, PhD (H. Lee Moffitt) discussed strategies for obtaining the K99/R00 Pathway to Independence Award (http://grants.nih.gov/grants/guide/pa-files/PA-11-197.html). This grant is unique in that it offers up to 2 years of mentored research time at the candidate’s institution (K99 phase), and then the candidate switches to an independent faculty position at their present, or another, institution for up to 3 years (R00 phase). It is the only K award that does not have a U.S. citizenship/permanent residency requirement. The candidate cannot have completed more than 5 years of postdoctoral research, have had a prior K or R level award, or be in an independent research position. The main part of the grant is divided into a Candidate Information section and a Research Strategy section. Additional components include a description of the Research Environment, an Institutional Commitment letter, letters of support from a mentor and/or co-mentors, and training in the responsible conduct of research. Dr. Pilon-Thomas emphasized the importance of having a strong mentor who is not just a supervisor, but who is available to help you develop as a scientist and has a track record of training postdoctoral fellows [1]. She recommended describing the mentor’s research and how it both synergizes and differs from the candidate’s proposal. The proposal also needs a means of evaluating the candidate’s progress. The Research Strategy should summarize how the mentored phase will be important for the independent phase, and show evidence of long-term viability. For grants targeted to the National Cancer Institute, the proposal must include work on human tissues or human cancers. Thus the K99/R00 is an important mechanism for those scientists looking to enter academics, not only to establish a track record in getting funding, but to provide a means to have support for mentored training followed by an independent position.

### Getting a K08 award from the NIH

Chris Gamper, MD, PhD (Johns Hopkins University) presented strategies for getting a K08 Mentored Clinical Scientist Award (http://grants.nih.gov/grants/guide/pa-files/PA-11-193.html). This grant offers 3–5 years of protected salary and supply support as mentored research time for individuals with clinical doctoral degrees. Candidates must not have had a prior K or R level award, and cannot be in an independent research position. The critical components of the application include Candidate Information, a Career Development Plan, Mentor letter, and a Research Plan. Dr. Gamper recommended stating your short and long-term career goals, as well as your desire to be an independent researcher. Importantly, he stated that the K08 is funding your training, not just your research plan. He stressed the importance of showing how the candidate’s goals are distinct from your mentor’s goals. Dr. Gamper also recommended taking stock of your deficiencies, and how you plan to correct them in your training plan and outlining how clinical responsibilities will be balanced with the basic research component is also critical. Regarding the mentor, he thought having an expert in the same field as the proposed field of study that is accessible to the candidate and has a track record of mentoring would be best. A co-mentor may be appropriate, even from another institution, if they have expertise that complements the primary mentor background in the chosen field of study. The Research Plan should have 2–4 aims, and the aims should “stand alone” and not be dependent on the outcomes of other aims. For resubmissions, respond to all of the reviewers’ comments, consider adding additional expertise as co-mentors and demonstrate productivity since the prior submission [2].

### New primary investigators: attracting students and post-docs, preparing for tenure

Weiping Zou, MD, PhD (University of Michigan) explained how to build and lead a research team, and prepare for tenure. He emphasized that success depends on hiring the right people and keeping them motivated to empower them to do their best work [3]. Before hiring, the Primary Investigator (PI) must know their own strengths, their projects, technical skills that they lack, needed level of training in their staff, and the funding available to support the team. Advertising open positions both by informal discussion with mentors and colleagues as well as through conferences and journals is critical. When hiring, be sure to call references, understand the candidate’s career goals and make sure the candidate impresses you in an interview. As motivation to the team, Dr. Zou recommends having interesting projects, regular lab meetings and personal mentoring [1]. He reminded the group that graduate students and postdocs eventually become your colleagues, scientific partners and collaborators, so treat them well. Regarding preparation for tenure, he suggested obtaining general information on the academic pathways at your institution, having a junior faculty committee with 4–6 senior faculty members, and listening to recommendations that lead to promotion. The faculty manual should have information regarding the average length needed for tenure, promotion criteria and organizational structure. Dr. Zou emphasized that networking both within and outside your institution is critical, and that success is a combination of knowing how to focus, obtain funding, work well with others and balance your time.

### The business of research

Lisa Butterfield, PhD (University of Pittsburgh) presented strategies to leading a successful laboratory. First she suggested emulating those labs that are successful and avoiding the pitfalls of labs that are in bad situations. She recommended becoming an active member of your institution, including participation in committees, giving seminars and reviewing grants. Dr. Butterfield suggested applying for funding from any potential source interested in your research, presenting your data frequently and incorporating reviewers’ feedback. Regarding grants, she recommended keeping your CV up-to-date in real time, reading other successful grants and having someone help you review comments. Utilizing career training workshops and internal grant review boards are always helpful. She also recommended publishing frequently in as appropriate and high impact journals as possible, and making sure at least 33% of publication are first or last author. She explained how hiring talented staff and providing them with a good work environment will lead to success. Dr. Butterfield suggested speaking with references by phone before interviewing staff candidates, and to be the best mentor you can be by setting clear expectations and acting professionally at all times. She recommended tracking your spending to stay within budget, but not to be afraid to spend in order to create more opportunities to earn better grants.

### Career panel

Ana Anderson, PhD (Harvard) discussed her career path into academia, including the challenges of work-life balance, underperforming employees and finding good mentors. She outlined 5 essential elements for good career building: commitment, organization, mentorship, information and communication. She recommended learning how to cope with grant/manuscript rejection, working long hours and weekends. Strategies that help you organize your work flow, personnel and personal life are critical. She emphasized the importance of finding a good mentor, such as someone who is accessible, an advocate and has a life-long commitment. Dr. Anderson informed the audience that each institution has information that outlines criteria for promotion, hiring and grants administration. Regarding communication, she recommended giving oral presentations, writing primary research and review papers, collaborating and getting feedback from mentors.

Amy Wesa, PhD (CelSense, Inc) explained which career paths the PhD biomedical workforce enters after training is completed, and that about 18% end up in industry, including companies that produce drugs, devices or life science tools. There are also contract research organizations that perform discovery, preclinical or clinical studies for a fee. Within an organization, there are different career ladders that can be navigated, which will vary by work environment. For example, Celsense, Inc. is a privately held small biotechnology company offering novel imaging agents to monitor cells by magnetic resonance imaging. Working in a small company means employees perform multiple roles, which can include marketing, fundraising, research, customer support and regulatory compliance. At smaller companies, research tends to be on a smaller scale and customer-driven. She also discussed how they bring products to market in terms of having good manufacturing practices, quality control studies, preclinical studies and good laboratory practice studies. Candidates should be willing to develop their writing skills, be comfortable giving presentations to scientific and lay audiences, and be willing to network. The major distinctions from academia include that everyone in the company is working toward a common goal, whereas each lab at an academic institution has different goals. There are not typically students in a company, there is more team recognition and success is not defined by publications.

Raj Puri, MD, PhD (Food and Drug Administration) presented the general organization of the FDA and how the Center for Biologic Evaluation and Research (CBER) regulates biological products. He explained that CBER does conduct some research to stay ahead of the curve as products and technologies evolve. Since products and regulatory paradigms constantly evolve, cutting edge research at CBER helps prepare the way for anticipated products and develop appropriate policy. He discussed the current research areas as well as types of positions available in the FDA. Dr. Puri also described the fellowships available to provide training in regulatory review, including the Interagency Oncology Task Force Fellowship and the FDA Commissioner’s Fellowship Program. He discussed the responsibilities of regulatory reviewers and PIs in product review, policy development, outreach and research.

## Conclusions

The SITC Professional Development Session provided information relevant to early career scientists looking to succeed in a career in cancer immunotherapy. Advice was geared toward graduate, medical and post baccalaureate students as well as postdoctoral fellows, clinical fellows and Assistant Professors. Overriding themes included being exceptionally organized and enthusiastic when preparing a grant application. Identifying a great mentor was critical both in preparing grants, getting your first job and in maintaining success as an early career professional. The field of cancer immunotherapy has clear opportunities in academia, industry or government, and knowing how each environment operates is critical in matching your scientific skills sets with the right career path.

For more information, the session was recorded and can be viewed at http://www.sitcancer.org/sitc-meetings/presentations#.

## Competing interests

The authors declare that they have no competing interests.

## Authors’ contributions

CMC, WLR and KASW drafted and revised the manuscript. All authors read and approved the final manuscript.
